# Expulsion Large Uric Calculus

**Published:** 1874-02

**Authors:** John E. Walker


### EXPULSION OF LARGE URIC CALCULUS.

BY JOHN E. WALKER, M.D.

EDITOR ATLANTA MEDICAL AND SURGICAL JOURNAL:—In compliance with your request, I furnish you for publication a report of my own case.

Since 1849, at intervals of five or six years, I have been a sufferer from renal calculi, which have usually passed the ureters in two or three days, in one or two attacks, in a few hours after, I felt pain in the kidney; and have generally passed out of the bladder in one or two days after reaching that viscus. I, however, a few years since, discharged one, the presence of which I had never suspected until it lodged in the urethra, during micturition. It was large and quite rough, and I think could not have been in the bladder for any length of time. I suppose it must have passed the uterer during sleep.

In August, 1872, I was attacked with the usual symptoms of stone in the kidney, the right side, which, after troubling me at intervals for two or three weeks, entirely ceased. Under the use of powerful relaxants, such as hot baths, emetics, and a large bleeding, I had no further trouble, not a symptom, for the space of twelve months. On the 17th of August last I again felt pain, which I at once recognized, and after three hours of great agony, felt the stone pass into the bladder. Trusting, as I had done in former instances, that it would soon pass out, I gave myself no concern for several days. Finding that it would not, by the natural efforts, and that it was producing some irritation at the neck of the bladder, and giving me pain after micturition, I began treatment by the introduction of bougies, beginning with one of small size and gradually increasing, until I could easily introduce a No. 11 metallic instrument. Invariably the urine would pass out along side of the bougie; spasm at the neck would occur, so that my hopes of having the stone follow the instrument as it was withdrawn were entirely disappointed. After repeated failures, and much irritation, I abandoned that course, and contrived a noose of horse-hair, which was attached to a wire. This I introduced through the canula of Lallemand's *porte caustique;* the hair, from its elasticity, would spread out like a net, and I hoped to engage the stone in its meshes, and by gentle traction, to overcome the spasm, and draw it out. I could not suppose it to be large, as it had so recently passed the ureter. In this novel operation I was disappointed; the irritation was so great that I could not bear the presence of the instrument in the bladder long enough to engage the stone. I intended if I should find the stone too large, to be drawn out in this manner, to attempt to crush it with the mouth of the canula by traction on the wire to which the hair was attacked; failing in this, I would disengage the stone and withdraw the instrument. After these repeated failures, I decided in my own mind that I could not be relieved without crushing. I accordingly procured from my friend, Prof. H. F. Campbell, of Augusta, a lithrotrite, and undertook the operation on myself, but the irritation at the neck of the bladder had become so great that I could not bear the introduction of the instrument. After this I made no further effort, but determined to seek the advice of wise and experienced surgeons, and to submit to whatever treatment they would decide upon. My friend and relative, Dr. Hitchcock, of your city, invited me to Atlanta, and offered me the hospitalities of his house. His interest and sympathy for me induced him to call on you and ask your advice. You, dear Doctor, though personally a stranger, had the kindness to write me an encouraging letter, replete with practical suggestions. Upon this I determined to try again. On the night of the 24th ult. I introduced a No. 11 metallic bougie, but as in all previous trials, the urine, which had been purposely allowed to accumulate, flowed past the instrument before it entered the bladder; spasm of the neck came on, so that the stone could not follow the instrument as it was withdrawn. I determined not to give it up, but reintroduced the bougie; prostrated myself on the knees and elbows, and made violent straining efforts as I withdrew the instrument, but all seemed to be vain. On the next day I took freely of Risley's Buchu, not having the Lithia, which you kindly suggested, at hand. Towards evening I retained the urine until the bladder was well filled; at night I rubbed the perineum with extract of belladonna, and introduced a small quantity of the same into the rectum. After allowing sufficient time for its effect, I armed the bougie, No. 11, with the extract, and, after lubricating it well with castile soap, I introduced it very carefully and slowly until it reached the neck of the bladder; then waited to overcome the spasm, but pressing gently. All of a sudden the urine gushed out with such force as to drive the instrument before it clear of the urethra. I felt relieved at the neck of the bladder, and supposed that the stone had passed after the bougie. I made a careful search for it on the floor, but not finding it, I decided to reintroduce the instrument and try again. On attempting to do so, I discovered the pointed extremity of the calculus just within the meatus, and with a probe and a little coaxing, easily extracted it. It is shaped like a bean, seven-sixteenths by five-sixteenths of an inch in its diameters, is of a dark brown color, very rough at its extremities. It is of the mixed or alternating class. Since its passage I have felt some vesical irritation, which the citrate of lithia, has promptly relieved. My mind has been freed from a great burden of anxiety; my body from much pain and suffering.

I sincerely trust that the recital of my case, and the manner in which I have been happily relieved, may aid my brethren of the profession in relieving others similarly afflicted. I have met with not less than a score of cases in my practice, which have all been relieved without crushing or cutting, except one, a boy six years old, who had a large calculus, was subjected to lithotomy by Prof. Dugas, at my request, during the war. The operation was entirely successful; he has had no difficulty since, and is now a stout, healthy young man.

There is one point of special interest in my case. The stone recently passed must be the same that troubled me last August, a year ago, which for twelve months I never felt, although engaged in an active practice, spending at least half my time in a buggy and on horseback, over rough and rugged country roads. It may be possible, though I think not probable, that the stone felt in 1872 reached the bladder, and was dissolved or disintegrated, and passed away in particles.

To you, my dear Doctor, I am greatly indebted for the happy relief I have experienced. Encouraged by the confident tone of your sound advice, I renewed my efforts, and complete deliverance has been the result.

